# Synchronization of entorhinal cortex stellate cells

**DOI:** 10.1186/1471-2202-13-S1-P167

**Published:** 2012-07-16

**Authors:** Patrick Crotty, Eric Lasker, Sen Cheng

**Affiliations:** 1 Department of Physics and Astronomy, Colgate University, Hamilton, NY 13346, USA; 2 Mercator Research Group 1, Fakultät für Psychologie, Ruhr-Universität Bochum, 44801 Bochum, Germany

## 

Synchronized oscillations of large numbers of central neurons are believed to be important for a wide variety of cognitive functions, including long-term memory recall and spatial navigation. It is therefore plausible that evolution has optimized the biophysical properties of central neurons in some way for synchronized oscillations to occur.

The stellate cells in layer II of the entorhinal cortex are involved in the representation of positional information through their role as “grid cells” projecting to the place cells of the hippocampus [1]. Both place cells and grid cells exhibit a large scale synchronized background oscillation in the theta range (8 to 12 Hz). This background oscillation is believed to be necessary for the phase coding of location within a place or grid field [2].

We used computational models of these cells [3] to investigate the relationships between the presumably genetically determined parameters of stellate cells in layer II of the entorhinal cortex and the ease with which coupled populations of these cells synchronize their intrinsic oscillations: in particular, we calculated the time it takes cells with initially randomly distributed phases to synchronize their oscillations to within one action potential width, and the metabolic energy they consume in doing so. The parameters we varied were the maximum conductances for the persistent sodium current and the hyperpolarization activated cation current, which have the most effect on the intrinsic firing frequency of the neurons.

**Figure 1:**
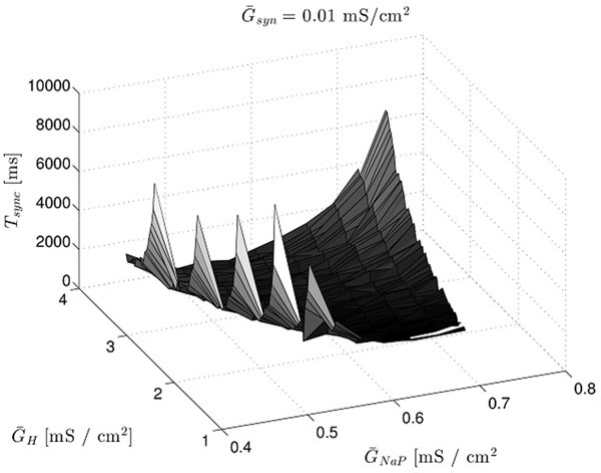
The mean time for 3 stellate cells with all-to-all inhibitory coupling to synchronize their firing from an initially random distribution of phases is shown as a function of the maximum conductances for the persistent sodium current and the hyperpolarization activated cation current. The valley of minima is in a region of parameter space corresponding to the theta frequency.

We found that the 8 to 12 Hz intrinsic firing frequency range which has been observed for these cells is strongly advantageous for both synchronization time and metabolic energy consumption. Changing the conductances so as to make the frequency either higher or lower results in a notable increase in the time it takes the coupled stellate cells to synchronize their oscillations. This optimization (see figure 1) appears to be largely independent of the number of cells, the network topology, and whether the coupling is excitatory, inhibitory, or heterogeneous. The metabolic energy consumption shows a similar minimum in this frequency range. The implication is that the theta frequency is preferred as the background frequency in the entorhinal cortex because of the synchronization time advantage.
